# Advocating for rigorous and multifactorial analyses in post-COVID cognitive research

**DOI:** 10.1016/j.lanwpc.2024.101166

**Published:** 2024-08-03

**Authors:** Qingjia Zeng, Dan Shan

**Affiliations:** a Institute of Medical Information/Medical Library, Chinese Academy of Medical Sciences, Peking Union Medical College, Beijing, 100020, China; b Department of Biobehavioral Sciences, Columbia University, New York, NY, 10027, USA

To the Editor,

We read with interest the article titled “Brain abnormalities in survivors of COVID-19 after 2-year recovery: a functional MRI study” by Zhao et al. and commend their findings.^1^ However, several areas warrant further discussion and exploration, which are crucial for future research directions in this field.

Most importantly, although the authors acknowledged the limitation of not assessing cognitive function of COVID-19 survivors prior to their COVID-19 infection, understanding the baseline cognitive state of survivors before infection, compared to healthy controls, is indispensable for several reasons. First, survivors with poorer initial cognitive function can be more susceptible to infection, possibly due to difficulties in understanding reliable preventive measures. Second, lacking sufficient self-protective awareness (i.e., difficulty adhering to COVID-19 prevention guidelines) also makes them more likely to suffer COVID-19 and repetitive infections. Third, these individuals may find it challenging to follow rehabilitation strategies post-infection. Fourth, there is a potential for selection bias in the sample (i.e., non-representative COVID-19 survivors). Therefore, we highlight the importance of considering these factors in future research. Addressing this limitation might be effectively achieved through utilizing the propensity score matching approach.

While the authors matched control participants for age, sex, and education level, they did not consider income and employment status, which significantly influence mental health. Both low income and unemployment are linked to increased rates of emotional disorders. In addition, during multiple periods of the study, China enforced stringent quarantine and isolation policies, which are associated with cognitive impairments and adverse psychological impacts.^2^^,^^3^ Considering these sociodemographic factors and their association with brain abnormalities is crucial to mitigate the risk of confounding variables.

Furthermore, the authors divided COVID-19 survivors into mild-moderate and severe-critical groups based on the severity of their initial illness. However, they did not specify whether participants had experienced multiple COVID-19 infections, which could confound the results. Repeated infections might exacerbate cognitive impairments and psychiatric symptoms. Additionally, while they collected information on COVID-19 vaccination status, they did not indicate whether they controlled for this confounding factor in their statistical analyses. This oversight is significant, as vaccination can significantly improve prognosis after COVID-19 infection.^4^ Therefore, conducting subgroup analyses based on the number of COVID-19 infections and vaccination status (never vs. single dose vs. multiple doses) would provide more granular insights into their cognitive impacts.

Lastly, while the authors identified cognitive complaints, psychiatric, and neurological symptoms among COVID-19 survivors, they did not explore the potential causal relationships between these conditions. Future research may include mediation analyses to disentangle these effects and determine whether their cognitive impairments are a direct result of COVID-19 virus or secondary to psychiatric conditions induced or exacerbated by the pandemic consequences (e.g., stress, anxiety, quarantine, and social isolation). Determining these relationships could have implications for psychological and social support interventions, in addition to medical treatments targeting the virus itself.

Despite these limitations, a recent meta-analysis has suggested that COVID-19 infection could significantly increase the risk of developing new-onset dementia.^5^ Thus, Zhao et al. have provided valuable findings through fMRI data, offering more objective evidence beyond cognitive function scales to support the potential association between COVID-19 infection and an increased risk of NOD. Given the irreversible nature and profoundly damaging effects of dementia on individuals, families, and society, Zhao et al.'s neuroimaging findings provide potential targets for treatment and intervention in individuals experiencing brain fog from long-COVID.^1^

## Contributors

Qingjia Zeng: Study design & Writing the manuscript draft.

Dan Shan: Study design & Manuscript revision.

## Declaration of interests

We declare no competing interests.
